# Supplementary material for: A bioengineered niche promotes in vivo engraftment and maturation of pluripotent stem cell derived human lung organoids
Source: eLife. 2016 Sep 28;5:e19732. doi: 10.7554/eLife.19732 (PMC5089859; doi:10.7554/eLife.19732)
Supplement: Figure 1—source data 1. — The number of ECAD+ structures were counted and scored as positive or negative for NKX2.1 expression. The percent NKX2.1+ epithelial structures were calculated (NKX2.1+ECAD+/total ECAD+) for each tHLO (conditions listed). The averages are listed in the bottom row. DOI: http://dx.doi.org/10.7554/eLife.19732.004 [file elife-19732-fig1-data1.docx]

| **Condition** | **Total # of epithelial structures per cross section** | **# epithelial structures NKX2.1+** | **Percent NKX2.1+ epithelial structures** |
| --- | --- | --- | --- |
| FGF10 Matrigel | 10 | 7 | 70.00% |
| FGF10 Matrigel | 1 | 1 | 100.00% |
| FGF10 Matrigel | 57 | 46 | 80.70% |
| FGF10 Matrigel | 39 | 26 | 66.66% |
| FGF10 Matrigel | 5 | 5 | 100.00% |
| FGF10 Matrigel | 30 | 30 | 100.00% |
| FGF10 Matrigel | 3 | 3 | 100.00% |
| No Matrigel | 29 | 25 | 86.21% |
| No Matrigel | 31 | 24 | 77.42% |
| No Matrigel | 21 | 17 | 80.95% |
| **Average** | **22.6** | **18.4** | **86.19%**  **SEM: 4.14%** |

Figure 1- source data 1
