# Supplementary material for: A bioengineered niche promotes in vivo engraftment and maturation of pluripotent stem cell derived human lung organoids
Source: eLife. 2016 Sep 28;5:e19732. doi: 10.7554/eLife.19732 (PMC5089859; doi:10.7554/eLife.19732)
Supplement: Figure 2—source data 1. — The number of NKX2.1+ airway-like structures were counted that also possessed ACTTUB+ multiciliated cells, CC10+ secretory cells, or MUC5AC+ secretory cells. The percent of airways possessing each cell type was calculated (ACTTUB+NKX2.1+/Total NKX2.1+ epithelial structures) for each tHLO (conditions listed). This same equation was applied for CC10+ and MUC5AC+ secretory cells. The averages are listed in the bottom row. DOI: http://dx.doi.org/10.7554/eLife.19732.011 [file elife-19732-fig2-data1.docx]

| **Condition** | **# NKX2.1+ Airway-like structures that contain Multi-ciliated cells (ACTTUB)** | **Total # of NKX2.1+ Airway-like structures per cross section** | **Percent of Airway-like Structures with Multi-ciliated cells (ACTTUB)** |
| --- | --- | --- | --- |
| FGF10 Matrigel | 5 | 6 | 83.33% |
| FGF10 Matrigel | 10 | 10 | 100.00% |
| FGF10 Matrigel | 3 | 3 | 100.00% |
| FGF10 Matrigel | 17 | 18 | 94.44% |
| FGF10 Matrigel | 1 | 1 | 100.00% |
| FGF10 Matrigel | 6 | 6 | 100.00% |
| No Matrigel | 9 | 9 | 100.00% |
| No Matrigel | 1 | 1 | 100.00% |
| No Matrigel | 9 | 9 | 100.00% |
| **Average** | **6.78** | **7** | **97.53%**  **SEM: 1.88%** |
| **Condition** | **# NKX2.1+ Airway-like structures that contain CC10+ (Club) Cells** | **Total # of NKX2.1+ Airway-like structures per cross section** | **Percent of Airway-like Structures with CC10+ (Club) Cells** |
| FGF10 Matrigel | 0 | 2 | 0.00% |
| FGF10 Matrigel | 6 | 14 | 42.86% |
| FGF10 Matrigel | 2 | 6 | 33.33% |
| FGF10 Matrigel | 4 | 18 | 22.22% |
| FGF10 Matrigel | 0 | 6 | 0.00% |
| FGF10 Matrigel | 9 | 10 | 90.00% |
| No Matrigel | 8 | 13 | 61.54% |
| No Matrigel | 1 | 1 | 100.00% |
| No Matrigel | 9 | 15 | 60.00% |
| **Average** | **4.33** | **9.44** | **45.55%**  **SEM: 11.93%** |
| **Condition** | **# NKX2.1+ Airway-like structures that contain MUC5AC+ (Goblet) Cells** | **Total # of NKX2.1+ Airway-like structures per cross section** | **Percent of Airway-like Structures with MUC5AC (Goblet) Cells** |
| FGF10 Matrigel | 0 | 2 | 0.00% |
| FGF10 Matrigel | 8 | 14 | 57.14% |
| FGF10 Matrigel | 3 | 6 | 50.00% |
| FGF10 Matrigel | 11 | 18 | 61.11% |
| FGF10 Matrigel | 6 | 6 | 100.00% |
| FGF10 Matrigel | 10 | 10 | 100.00% |
| No Matrigel | 12 | 13 | 92.31% |
| No Matrigel | 1 | 1 | 100.00% |
| No Matrigel | 11 | 15 | 73.33% |
| **Average** | **6.89** | **9.44** | **70.43%**  **SEM: 11.02%** |

Figure 2- source data 1
